# Special Issue: Advances in Chemical Vapor Deposition

**DOI:** 10.3390/ma13184167

**Published:** 2020-09-19

**Authors:** Dimitra Vernardou

**Affiliations:** 1 Department of Electrical and Computer Engineering, School of Engineering, Hellenic Mediterranean University, 710 04 Heraklion, Greece; dvernardou@hmu.gr; Tel.: +30-2810-379631; 2 Center of Materials Technology and Photonics, School of Engineering, Hellenic Mediterranean University, 710 04 Heraklion, Greece

**Keywords:** CVD, electrochromism, perovskite photovoltaic materials, TiO_2_, Al_2_O_3_, VO_2_, V_2_O_5_, computational fluid dynamics

## Abstract

Pursuing a scalable production methodology for materials and advancing it from the laboratory to industry is beneficial to novel daily-life applications. From this perspective, chemical vapor deposition (CVD) offers a compromise between efficiency, controllability, tunability and excellent run-to-run repeatability in the coverage of monolayer on substrates. Hence, CVD meets all the requirements for industrialization in basically everything including polymer coatings, metals, water-filtration systems, solar cells and so on. The Special Issue “Advances in Chemical Vapor Deposition” has been dedicated to giving an overview of the latest experimental findings and identifying the growth parameters and characteristics of perovskites, TiO_2_, Al_2_O_3_, VO_2_ and V_2_O_5_ with desired qualities for potentially useful devices.

In a Chemical Vapor Deposition (CVD) process, the reactants are transported to the substrate surface in the form of vapors and gases. Although there are exceptions, the vapor of the reactive compound, usually an easily volatilized liquid or in some cases a solid, would sublime directly and is generally prepared by injection of the liquid into solvent or heated evaporators [1]. The vapor is then transported to the reaction zone by a carrier gas. The unwanted gas phase nucleation (homogeneous reaction) in CVD can be eliminated through high carrier-gas flow rates, minimum temperatures and cold wall reactors [2].

Would it be possible to assemble nanostructures with confined atomic level thickness, high specific surface area and outstanding surface chemical states at large scale and low cost? CVD is compatible with in-line manufacturing processes where material properties can be controlled with great accuracy, varying growth parameters such as temperature, precursor composition and flow rate. There are various CVD technologies including pulsed-pressure metal organic CVD, atmospheric pressure CVD, atomic layer deposition, spray pyrolysis, plasma-enhanced CVD, aerosol-assisted CVD and so on. There are so many variations on CVD technology because there is no possibility of direct control of the basic processes occurring at the deposition surface. Some of the process technologies that influence the materials’ basic characteristics and, as a consequence, their potential application, are included in this Special Issue. The review article by Liu et al. [3] reported on the perovskite photovoltaic materials, with an emphasis on their development through CVD to deal with challenges such as stability, repeatability and large area fabrication methods. In this article, one can gain a clear picture of the influence of different CVD technologies and how the experimental parameters can optimize the perovskite materials for the respective devices.

Pulsed-pressure metal organic CVD (PP-MOCVD) can be utilized for the development of low-cost coatings with both macro and micro-scale, three-dimensional features. Films such as TiO_2_ can be uniformly deposited with control of the nanostructure dimension and the coating thickness [4]. Towards this direction, Gorthy et al. [5] highlighted the urgent need for anti-microbial coatings due to the pandemic of COVID-19 through the growth of nanostructured TiO_2_ onto handles, push-plates and switches in hospitals. The morphology nanocharacteristic is believed to be the key function for photocatalytic activity with enhanced durability.

CVD at atmospheric pressure (APCVD) is a thin film deposition process with typically high deposition rates. It is an attractive method because it was designed to be compatible with industrial requirements (up-scaling at low cost and high process speed) [6]. The optimization of APCVD towards the development of high yield processes can result in the excellent controllability of the materials’ stochiometry, isolating different polymorphs of VO_2_ [7]. Among the various polymorphs of VO_2_, only the monoclinic VO_2_ is a typical thermochromic material [7]. In particular, it is known to undergo a reversible metal-to-semiconductor transition associated with a transformation from monoclinic to tetragonal phase at a critical temperature [8]. Therefore, the utilization of a simple, low cost process with up-scalable possibilities for the development of VO_2_ coatings in thermochromic windows is a priority. In the review paper of Drosos et al. [1], the progress on experimental procedures for isolating different polymorphs of VO_2_ is outlined. Additionally, the importance of understanding and optimizing the behaviour of the materials supported by modelling studies is highlighted. In that way, theory meets practice, whereas cross-check procedures take place in order to establish firm materials with advanced characteristics.

Atomic layer deposition (ALD) is a process based on the gas phase chemical process in a sequential manner. The majority of ALD processes occur at temperatures > 100 °C with an exception of Al_2_O_3_. In particular, it can be accomplished with a variety of precursors, in relatively short times and at low temperature [9]. Xia et al. [10] reported the potential to grow Al_2_O_3_ at 200 °C utilizing different Al precursors via ALD. A consistent 0.12 nm/cycle on glass, Si and quartz substrates was demonstrated to give complex nanostructures with conformity, uniformity and good thickness control as a protection layer in photoelectrochemical water splitting.

Spray pyrolysis is a process in which a precursor solution is atomized through a generating apparatus, evaporated in a heated reactor and decomposed on the top of the substrate into particles and thin films [11]. It is proven to be very useful for the preparation and the design of functional and versatile classes of materials at low cost and easy processing. This process can result in materials with enhanced electrochemical performance for electrochromic applications combined in layered and composite forms for higher reflective property, electrochemical stability and faster electrochromic response [12,13]. In Mouratis et al.’s letter [14], a new approach regarding the development of V_2_O_5_ electrochromic thin films at 250 °C using ammonium metavanadate in water as precursor is shown. The precursor concentration can affect the morphology of the oxides, resulting in a large active surface area suitable for electrochromic applications.
